# Dynamics of Responses in Compatible Potato - *Potato virus Y* Interaction Are Modulated by Salicylic Acid

**DOI:** 10.1371/journal.pone.0029009

**Published:** 2011-12-14

**Authors:** Špela Baebler, Katja Stare, Maja Kovač, Andrej Blejec, Nina Prezelj, Tjaša Stare, Polona Kogovšek, Maruša Pompe-Novak, Sabine Rosahl, Maja Ravnikar, Kristina Gruden

**Affiliations:** 1 Department for Biotechnology and Systems Biology, National Institute of Biology, Ljubljana, Slovenia; 2 Department of Stress and Developmental Biology, Leibniz Institute of Plant Biochemistry, Halle (Saale), Germany; Max Planck Institute for Chemical Ecology, Germany

## Abstract

To investigate the dynamics of the potato – *Potato virus Y* (PVY) compatible interaction in relation to salicylic acid - controlled pathways we performed experiments using non-transgenic potato cv. Désirée, transgenic NahG-Désirée, cv. Igor and PVY^NTN^, the most aggressive strain of PVY. The importance of salicylic acid in viral multiplication and symptom development was confirmed by pronounced symptom development in NahG-Désirée, depleted in salicylic acid, and reversion of the effect after spraying with 2,6-dichloroisonicotinic acid (a salicylic acid - analogue). We have employed quantitative PCR for monitoring virus multiplication, as well as plant responses through expression of selected marker genes of photosynthetic activity, carbohydrate metabolism and the defence response. Viral multiplication was the slowest in inoculated potato of cv. Désirée, the only asymptomatic genotype in the study. The intensity of defence-related gene expression was much stronger in both sensitive genotypes (NahG-Désirée and cv. Igor) at the site of inoculation than in asymptomatic plants (cv. Désirée). Photosynthesis and carbohydrate metabolism gene expression differed between the symptomatic and asymptomatic phenotypes. The differential gene expression pattern of the two sensitive genotypes indicates that the outcome of the interaction does not rely simply on one regulatory component, but similar phenotypical features can result from distinct responses at the molecular level.

## Introduction

Potato (*Solanum tuberosum* L.) is the world's most widely grown tuber crop and the fourth largest food crop in terms of fresh produce after rice, wheat and corn. *Potato virus Y* (PVY), a member of the *Potyviridae* family, is an important potato pathogen worldwide. PVY^NTN^, belonging to the PVY^N^ strain group [1], is the most aggressive strain. In sensitive potato cultivars, PVY^NTN^ elicits the development of potato tuber necrotic ringspot disease, causing a decrease in the quality and quantity of potato production.

The ability of viruses to cause disease is determined by molecular interactions between the host plant and virus factors. These interactions directly affect virus replication and movement, symptom development and host defence responses [2]. Key signalling molecules in biotic interactions include salicylates, jasmonates and ethylene [3], but their specific roles depend on the particular host-pathogen interaction. Many studies have indicated that salicylic acid (SA; 2-hydroxybenzoic acid) is a key regulatory compound of disease resistance against fungi, bacteria and viruses (reviewed in [4]). SA has been shown to mediate resistance in many plant-virus interactions. Depending on the virus, SA can induce inhibition of viral replication and cell-to-cell or long distance viral movement (reviewed in [5]). Moreover, SA plays an important role in compatible interactions, where the basal level of SA mediates expression of a cohort of defence-related genes inducing a defence-like response [6]. Additionally, methyl salicylate appears to be the major communication signal for defence both within and between plants [7], [8].

The role of SA in the defence response in potato has not yet been thoroughly investigated. Potato plants contain high basal levels of SA [9]–[12], and their increase after fungal [9], [13] or viral [12] attack is rather moderate. Moreover, it has been shown that basal levels of SA in potato do not correlate with resistance to PVY^NTN^ [12].

The dynamics of plant-pathogen interactions are complex, and many processes can be misinterpreted if one only observes a snapshot of the interaction. Our previous studies have indicated that the timing of the response is crucial for the outcome of the interaction [14], [15]. Several studies of plant-virus interactions have shown that host gene expression responses vary drastically depending on time after viral infection [16]–[18].

To investigate the dynamics of the compatible plant-virus interaction in relation to SA-controlled pathways we have chosen three potato genotypes, differing in endogenous SA content and sensitivity to PVY^NTN^ infection. Potato plants of cv. Désirée allow multiplication of the virus both at the site of the inoculation as well as systemically, while developed symptoms of infection are very mild. This genotype was modified by expression of the *NahG* gene, encoding salicylate hydroxylase, which converts SA to catechol. NahG-Désirée was shown to accumulate SA in only minute amounts under various conditions [13], [19]. A third genotype, potato cv. Igor, which is highly sensitive to PVY^NTN^ infection, was included. In this genotype, necrotic and chlorotic spots appear on leaves a few days after infection, including wrinkles and mosaic chlorosis on non-inoculated leaves as the virus spreads, leading to a palm tree appearance [20]. Plants of the selected genotypes (cv. Désirée, NahG-Désirée and cv. Igor) were infected with PVY^NTN^. Multiplication and spread of the virus as well as expression of host genes that are markers for photosynthetic activity, carbohydrate metabolism and defence responses were followed in a time course experiment before and during symptom development. The results obtained indicate the involvement of SA as well as other signalling pathways in the potato-PVY interaction.

## Results

### SA-deficient potato plants develop strong PVY symptoms

**Figure 1:**
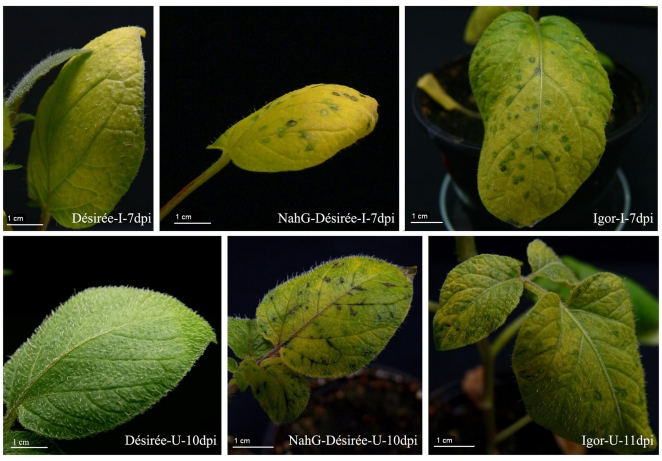
Symptom development following PVY inoculation in three potato genotypes. Local symptoms on inoculated (I) at 7 dpi and systemic symptoms on upper non-inoculated leaves (U) at 10 and 11 dpi of cv. Désirée, NahG-Désirée and cv. Igor.

To investigate the role of SA in the potato-PVY interaction, symptom development was monitored in genotypes differing in endogenous SA and sensitivity to virus (**Table S1**). In contrast to non-transgenic asymptomatic plants of cv. Désirée, in transgenic NahG-Désirée, which fail to accumulate SA [19], small round necrotic lesions were observed on inoculated leaves 5 days post inoculation (dpi) (**Figure 1**) followed by chloroses with green and necrotic spots. Systemic symptoms in the form of leaf vein necrosis appeared on NahG-Désirée 10 dpi (**Figure 1**) and became increasingly pronounced until the leaves fell off and a “palm tree” effect was observed. In contrast, no distinctive symptoms were observed in virus-inoculated compared to mock-inoculated plants of cv. Désirée (**Figure 1**), with the exception of faster yellowing of inoculated leaves.

The cv. Igor, which is highly sensitive to PVY^NTN^, showed first primary symptoms in parallel with NahG-Désirée at 5 dpi (**Figure 1**). However, observed local symptoms were more pronounced in NahG-Désirée compared to Igor plants. Systemic symptoms appeared in cv. Igor plants one day later than in NahG-Désirée plants, on 11 dpi (**Figure 1**), in the form of dark green rings with far less leaf vein necrosis in comparison to NahG-Désirée. As in NahG-Désirée the “palm tree” effect was also observed in cv. Igor.

Symptom appearance and disease progress were comparable in two independent transgenic NahG-Désirée lines (NahG-D2 and NahG-A) and can thus be considered independent from the position effect of *NahG* gene insertion. Therefore only one line, NahG-D2-Désirée, was used in further analyses of virus accumulation and gene expression.

### Faster onset of PVY multiplication at the site of inoculation in SA-deficient potato plants

**Figure 2:**
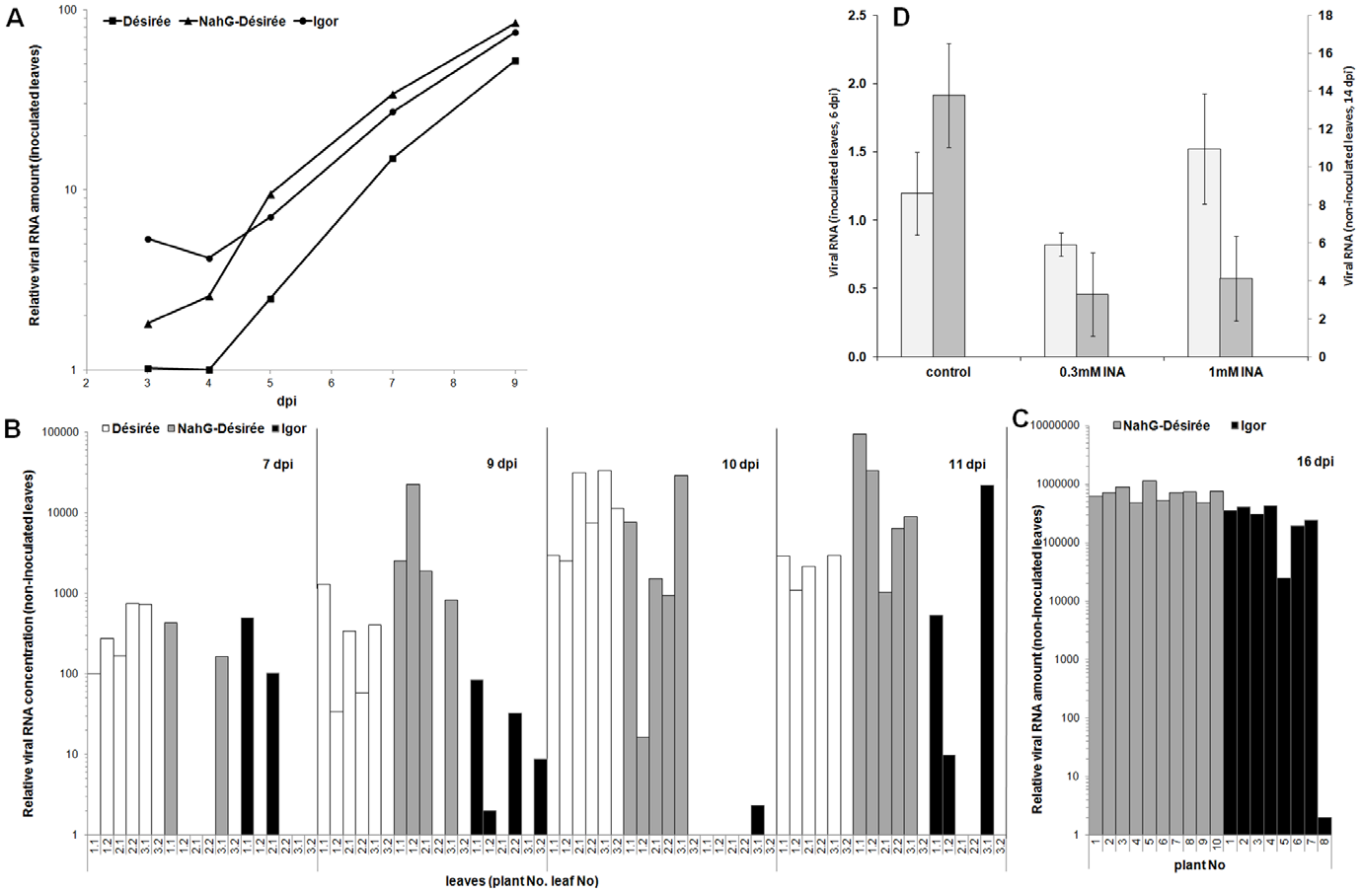
Accumulation of PVY RNA in leaves of potato plants. (A) Relative PVY RNA concentration (average of 3 individual plants) in inoculated leaves 3–9 days post inoculation (dpi). Results of statistical evaluation of data are shown in **Table 1**. (B) PVY RNA concentration (relative to the lowest detected viral amount) in non-inoculated leaves (3 plants per genotype, 2 leaves per plant: e.g. 1.2 denotes second leaf of plant 1) 7–11 dpi. (C) PVY RNA concentration in non-inoculated leaves of ten NahG-Désirée and eight cv. Igor plants at 16 dpi. PVY RNA concentrations are presented relative to the lowest detected viral amount in all plants. (D) Relative PVY RNA concentration (±SE, n = 4, normalised to COX gene expression) in the inoculated leaves 6 dpi (light grey) and in non-inoculated leaves 14 dpi (dark grey) of the NahG-Désirée plants following treatment with water (control), 0.3 mM or 1 mM INA.

Viral multiplication at the site of inoculation was measured in the investigated potato genotypes at 3, 4, 5, 7 and 9 dpi. At later time points viral RNA amount could not be measured because the inoculated leaves had fallen off. A slight increase in viral RNA accumulation above the residual inoculum level was first observed in NahG-Désirée at 4 dpi (**Figure 2A**), one day before symptom appearance. Because the residual inoculum levels were on average higher in cv. Igor, virus accumulation above the residual inoculum was not observed until 5 dpi. At 5 dpi, the viral RNA levels were significantly lower (p<0.05) in cv. Désirée than in the NahG-Désirée and cv. Igor genotypes, indicating delayed virus accumulation in the cv. Désirée. In the following days the kinetics of PVY RNA accumulation were similar in all three genotypes, with significant increase of viral RNA amount over time (**Table 1** **,** **Figure 2A**).

**Table 1:** Changes in expression of selected genes and viral RNA accumulation over time in different potato genotypes.

	Inoculated leaves									Upper non–inoculated leaves							
		Glu-I	Glu-II	Glu-III	PR-1b	CAB4	RA	GBSSI	PvyI		Glu-I	Glu-II	Glu-III	PR-1b	CAB4	RA	GBSSI
Désirée	dpi 4:3	•				−−				dpi 4:3	+						
	dpi 5:4		•			+	•		+++	dpi 5:4							
	dpi 7:5					•			+	dpi 7:5	−−		−				
	dpi 9:7					+++		+	+	dpi 9:7	−			•	++	+	•
										dpi 10:9	+++	++	+++	+++			•
										dpi 11:10							
	dpi 5:3		+	•	+				++	dpi 5:3	+						
	dpi 7:3		++	•	++	−−		•	+	dpi 7:3			•				
	dpi 9:3		+++	++	+++	•			++	dpi 9:3	−−		−		+++	++	
										dpi 10:3	+++	++	+++	+++	+++	•	+
										dpi 11:3	+++	+	+++	++	+++	•	++
NahG-Désirée	dpi 4:3					−−−		•	•	dpi 4:3	•				−−		•
	dpi 5:4				•	+			+	dpi 5:4		•					
	dpi 7:5	−−		•		−−−	−−−	−−−	•	dpi 7:5						−−	•
	dpi 9:7		+			+++		•	+	dpi 9:7					++		
										dpi 10:9	++		+				
										dpi 11:10							
	dpi 5:3	+	•	+	+	•		•	+	dpi 5:3							
	dpi 7:3	•			++	−−−	−−−	−−−	+	dpi 7:3					−−	−	−−
	dpi 9:3	−	++		++	−−	−−−	−−−	++	dpi 9:3				+		−	−
										dpi 10:3	++		+	+		−	−−
										dpi 11:3	++	•		•		−	−−
Igor	dpi 4:3				+++	−−−				dpi 4:3					−−−		
	dpi 5:4					++			+	dpi 5:4							+
	dpi 7:5				+	−−−	−−	−−−	+++	dpi 7:5		+	++	+	−−		
	dpi 9:7		−	−−		+++			+++	dpi 9:7	−	−−	−		+++	+	•
										dpi 10:9	+++				−	−−	−−−
										dpi 11:10	•				−−	•	
	dpi 5:3	+		+	+++	−−			+	dpi 5:3					−	+	+
	dpi 7:3	++	+	+	+++	−−−	−−	−−−	+++	dpi 7:3		+	+	•	−−−	++	++
	dpi 9:3	•			+++	−−	−−	−−	+++	dpi 9:3	−−				+++	+++	+++
										dpi 10:3			•		+	•	•
										dpi 11:3	++		+++	+			

The significance of increase (+++: p<0.001, ++: p<0.01, +: p<0.05, •: p<0.1) or decrease (---: p<0.001, --: p<0.01,-: p<0.05, •: p<0.1) in gene expression over time (PR-1b: pathogenesis-related protein 1b; Glu I,II,II: β-1,3-glucanase classes I, II, III; RA: RuBisCO activase; GBSSI: granule bound starch synthase I; CAB4: chlorophyll a-b binding protein 4) and viral accumulation (PvyI) is shown for comparisons between consecutive time points (upper panel) and to the first time point (3 dpi; lower panel; GBSSI in cv. Igor and cv. Désirée compared to 4 dpi). dpi 4:3 designates significance of difference in expression at 4 dpi versus 3 dpi. An empty field denotes no significance.

Systemic viral multiplication was measured on 3, 4, 5, 7, 9, 10 and 11 dpi. Two leaves from 3 independent plants were analysed per time point. Variation in viral RNA accumulation between individual plants of the same genotype was much higher in non-inoculated leaves than in the inoculated leaves, similarly as was observed in symptom appearance (**Table S1**). Therefore the analysis was insufficient for exact evaluation of viral spread kinetics; however, some trends were detected. Spread of viral RNA to upper non-inoculated leaves was first detected at 7 dpi in all genotypes, but only in plants of cv. Désirée viral RNA was present in the majority of analysed leaves in all time points (**Figure 2B**). In NahG-Désirée viral RNA was consistently detected from 9 dpi onward. In cv. Igor, however, the detection of PVY RNA in non-inoculated tissues was subject to extremely high variability, and altogether fewer than half of the tested leaves were positive. Therefore we analysed a new set of plants of cv. Igor at 16 dpi, when viral RNA was present in all analysed plants (**Figure 2C**).

The quantity of viral RNA was not time dependent in any of the investigated genotypes and was on average the highest in NahG-Désirée, followed by cv. Désirée (3-fold less), while in cv. Igor detected viral amounts were 2-fold lower than in Désirée (**Figure 2B**). Viral amount within each genotype was more uniform at16 dpi, but still showed greater variability and lower viral amounts in cv. Igor (**Figure 2C**).

### Pretreatment of NahG-Désirée with an SA analogue reverses phenotype resulting in low PVY symptom development

2,6-Dichloroisonicotinic acid (INA) induces a spectrum of defence responses similar to those of SA and is considered to be an analogue of SA. It is, however, not degradable by salicylate hydroxylase. To address the question of whether enhanced sensitivity of NahG-Désirée to PVY is indeed based on their inability to accumulate SA, a solution of 0.3 mM or 1 mM INA was sprayed onto leaves of NahG-Désirée (lines NahG-A and NahG-D2) 24 hours prior to their inoculation with PVY. For comparison, plants of the susceptible cv. Igor were sprayed with INA in parallel.

Primary symptoms were visible on inoculated leaves of most (4 out of 5) untreated NahG-Désirée 6 dpi (**Table S2**). Treatment with 0.3 mM INA and in particular with 1 mM INA delayed symptom appearance. The higher concentration retarded the symptom appearance by at least 2 days (**Table S2**). Treatment with INA also delayed yellowing of leaves. Systemic symptoms appeared at 12 dpi on upper intact leaves of untreated as well as on INA-treated NahG-Désirée transgenic lines; however the symptoms were less pronounced on the leaves of INA-treated plants, and the number of plants showing symptoms was smaller. In plants treated with 1 mM INA, pronounced systemic symptoms were observed as late as 18 dpi.

Viral RNA accumulation was measured at 6 dpi, at the time of appearance of primary symptoms on inoculated leaves of untreated NahG-D2-Désirée plants. Treatment with INA had no significant effect on viral RNA accumulation at the site of inoculation (**Figure 2D**), although in contrast to untreated plants, treated plants showed no local symptoms at this time point (**Table S2**). On the other hand, at 14 dpi when pronounced systemic symptoms were expressed on the upper non-inoculated leaves of untreated NahG-D2-Désirée plants, both concentrations of INA significantly (p<0.005) lowered the viral RNA accumulation, compared to control plants (**Figure 2D**), in correspondence with the absent or less-pronounced systemic symptoms observed in treated plants at this time point.

### Dynamics of plant responses at the site of inoculation

To better understand the observed differences in viral multiplication and spread in different potato genotypes, not directly related to kinetics of symptom development, we have investigated dynamics of host molecular responses. We have followed gene expression of pathogenesis-related (PR) proteins belonging to PR-1 and PR-2 families. PR-1b is a marker of early defence responses in potato [21], [22] that was shown to be upregulated 12 hours after PVY inoculation in an extremely resistant cultivar. Members of three classes of β-1,3-glucanases (Glu-I, II and III), belonging to PR-2 family, are known to be regulated in plant-viral interactions [23]. Moreover, all three classes were differentially expressed in susceptible genotypes following inoculation with PVY strains of different aggressiveness [15]. To indirectly follow regulation of photosynthesis and carbohydrate metabolism, we investigated expression of the chlorophyll a–b binding protein 4 (CAB4), RuBisCO activase (RA) and granule-bound starch synthase I (GBSSI) genes, previously shown to be involved in responses to PVY attack [15], [20]. Gene expression profiles of selected genes were measured in the virus-inoculated leaves 3, 4, 5, 7 and 9 dpi, before infected leaves fell off. To observe gene expression changes that are related only to virus inoculation, all levels of gene expression were normalized to expression in mock-inoculated samples. As another control, leaves were sampled before PVY inoculation (0 dpi). Results are presented in supporting information.

**Figure 3:**
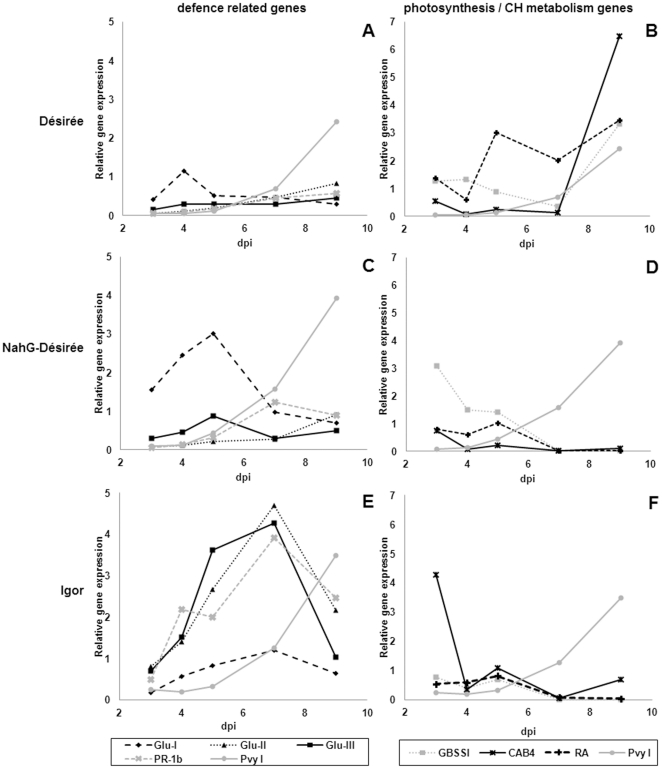
Dynamics of selected host gene expression in inoculated potato leaves. Expression of defence-related (A, C, E), photosynthesis and carbohydrate (CH) metabolism marker genes (B, D, F) in inoculated leaves of different potato genotypes (cv. Désirée, NahG-Désirée and cv. Igor) at 3, 4, 5, 7 and 9 days post infection (dpi). Relative viral RNA concentration (Pvy I) is plotted on each chart. PR-1b: pathogenesis-related protein 1b; Glu I, II, III: β-1,3-glucanase classes I, II, III; RA: RuBisCO activase; GBSSI: granule bound starch synthase I; CAB4: chlorophyll a–b binding protein 4. Data points represent the mean of three measurements. Statistical evaluation of data is shown separately in **Table 1**.

To gain insight into the expression pattern of selected marker genes, correlations in gene expression between individual host genes and between host gene expression and viral RNA concentrations over all time points and biological replicates within an individual genotype were first inspected. Two groups of highly correlated genes were identified in the inoculated leaves, corresponding to their physiological function, namely i) defence-related genes and ii) genes involved in photosynthesis and carbohydrate metabolism (**Figure S1A, Figure S2A**; lower left panels). In all three genotypes the highest correlation of gene expression was observed between the defence-related genes. The response of individual genes within this group was, however, genotype-specific. Analysis of dynamics of responses in inoculated leaves showed that the first responses on the level of gene expression precede the detected viral multiplication (**Figure 3** **, Figure S3**). The time course of expression of photosynthesis and carbohydrate metabolism marker genes in symptomatic plants (NahG-Désirée and Igor) is clearly distinct from the time course in asymptomatic plants (Désirée), where the activity of those genes recovers after the initial drop, whereas it stays low in symptomatic plants (**Figure 3B, D, F**). Interestingly, the response of defence-related genes was much stronger in both symptomatic genotypes. All investigated genes from that category showed strong induction in Igor plants. If comparing cv. Désirée with the NahG-Désirée genotype, a stronger response can be observed in the symptomatic NahG-Désirée (**Figure 3A, C**).

### Dynamics of plant responses in upper non-inoculated leaves

Evaluation of plant responses in the tissues that were not primarily infected was more difficult to explain because at several time points one replicate plant responded differently than the other two, as is clearly visible from the figure showing similarity of the gene expression profiles of individual plants (**Figure S1B, Figure S2B**). Inter-plant variability was especially pronounced in NahG-Désirée. However, it should be noted that gene expression in the outlier plants correlated better to the gene expression of plants collected at either previous or later time points (**Figure S1B**) indicating a difference in dynamics of responses in individual plants. Interestingly, the switch in expression can be observed already between 5 and 7 dpi in cv. Igor, while the first changes occur between 7 and 9 dpi in the Désirée and NahG-Désirée genotypes. Another, later switch of gene expression between 10 and 11 dpi was most pronounced in the Désirée genotype (**Figure S1B**).

We compared correlation of expression of individual genes in upper non-inoculated leaves. The response of defence-related genes was highly correlated in all genotypes (**Figure S1A, Figure S2A**, upper right panels). In the group of photosynthesis and carbohydrate metabolism genes, only CAB and RA are significantly correlated. Interestingly, no significant correlation was observed between the expression of individual genes and the viral RNA concentration in the same leaf.

**Figure 4:**
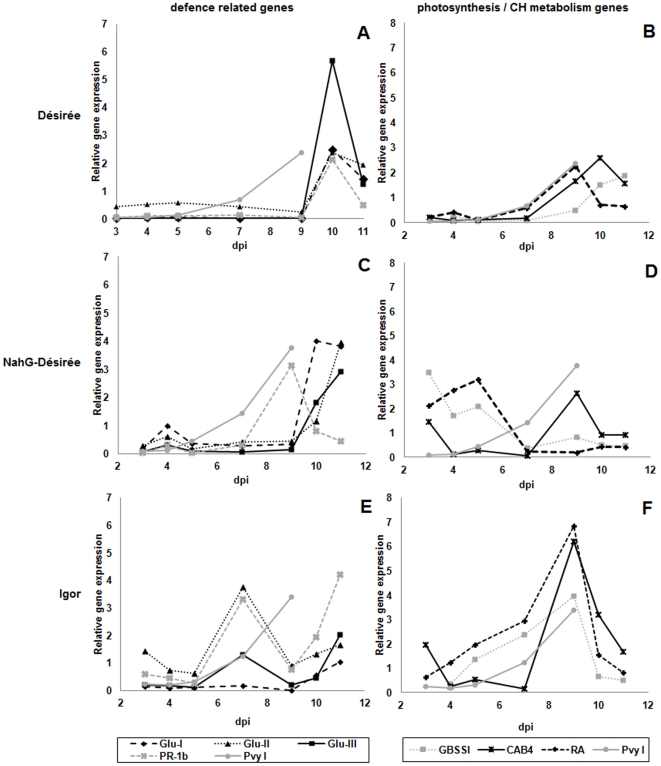
Dynamics of selected host gene expression in upper non-inoculated potato leaves. Expression of defence-related (A, C, E), photosynthesis and carbohydrate (CH) metabolism marker genes (B, D, F) in upper non-inoculated leaves of different potato genotypes (cv. Désirée, NahG-Désirée and cv. Igor) at 3, 4, 5, 7, 9, 10 and 11 days post infection (dpi). Relative viral RNA concentration (Pvy I) in the inoculated leaves is plotted on each chart. PR-1b: pathogenesis-related protein 1b; Glu I, II, III: β-1,3-glucanase classes I, II, III; RA: RuBisCO activase; GBSSI: granule bound starch synthase I; CAB4: chlorophyll a–b binding protein 4. Data points represent the mean of three measurements. Statistical evaluation of the data obtained is shown separately in **Table 1**.

As it was for the inoculated leaves, the time course of expression of individual genes was followed in upper non-inoculated leaves (**Figure 4**). In the cv. Désirée induction of photosynthesis- and carbohydrate metabolism-related genes occurred after 7 dpi (**Figure 4B**, **Table 1** **, Figure S4B**), coinciding with viral accumulation in non-inoculated tissues (**Figure 2B**). In symptomatic NahG-Désirée the transient increase of expression of RA and GBSSI at 5 dpi (**Figure 4D** **, Figure S4,** **Table 1**) coincides with the appearance of symptoms in the inoculated leaves. In Igor plants the most pronounced feature was a significant increase in CAB4, RA and GBSSI gene expression at 9 dpi (**Figure 4F** **,** **Table 1**), similar to, yet more intense, than in plants of cv. Désirée.

Interestingly, the response of defence-related genes in non-inoculated leaves differed between the two experiments performed. The most pronounced difference was in PR-1b expression. While in the first experiment a statistically significant response in PR-1b gene expression was detected in all three genotypes (**Figure 4** **,** **Table 1**), no significant differences in gene expression of PR-1b were observed in the second experiment (**Figure S4, Table S3**), either in mock or infected plants. The only defence-related gene responding similarly in non-inoculated leaves in both experiments was GluI. The responses were not correlated with either accumulation of viral RNA in the analysed tissue or to its physiological state.

## Discussion

The molecular mechanisms that underlie host physiological and phenotypic responses to virus infection are still largely unknown, although it has been shown that virus infection induces global activation or suppression of host gene expression [2], [14], [18], [20], [24], [25]. These gene expression changes reflect a combination of stress and defence-like responses, viral pathogenesis and host symptom development. The pathways controlling plant defence as well as viral pathogenesis are unique for the specific plant-virus interaction, mostly due to specificity of the virus' interactions with plant components and the extent of its multiplication in the plant (reviewed in [3]). Plants and viruses enter into various relationships that do not necessarily result in development of disease. In our study of compatible interaction all potato genotypes used allowed multiplication of the virus, although in cv. Désirée the symptoms were almost indiscernible (**Figure 1**), indicating the tolerant-like plant response of this genotype. On the other hand, depletion of SA in NahG-Désirée shifted the balance towards development of the disease. In particular, faster onset of virus multiplication (**Figure 2A**) and pronounced symptom development in NahG-Désirée was observed (**Figure 1**) indicating that the SA signalling pathway takes part in the tolerant-like response of the cv. Désirée. The role of SA in the compatible host-virus interaction was investigated previously in tobacco by SA treatment, which resulted in reduced viral accumulation and delayed appearance of the disease symptoms [26], [27]. Similarly, NahG potato lines showed decreased hypersensitive Nb-resistance-gene mediated response to *Potato virus X* (PVX) and were unable to induce systemic acquired resistance [28].

In our study, retardation of symptom appearance was observed in two NahG-Désirée lines after treatment with an SA-analogue (INA), confirming thus the role of SA in the exhibition of defence response. Despite causing a delay in symptom formation in NahG-Désirée, INA (at either concentration) did not decrease viral accumulation in the inoculated leaves at 6 dpi (**Figure 2D**). Possibly, the effect of INA was transient and occurred at earlier time points, as reported by Nie [27]. However, INA treatment resulted in reduced virus accumulation in non-inoculated leaves at 14 dpi (**Figure 2D**). In contrast to NahG-Désirée, INA treatment of the highly sensitive cv. Igor with a high basal level of SA, actually promoted symptoms (**Table S2**). Increased host sensitivity may have been the result of an *in planta* excessive concentration of SA and its analogues in the treated plants, as was shown in potato-*P. infestans* interaction [10].

To gain better insight into the mechanisms underlying the observed changes in disease outcome, we have followed both viral RNA multiplication and plant gene expression in a time-course experiment. The most pronounced differences between the two phenotypes (symptomatic and asymptomatic) were observed at 5 dpi, at the initial stage of viral multiplication and first development of local primary symptoms. Therefore we can conclude that the difference between viral RNA multiplication in symptomatic Igor and NahG-Désirée versus tolerant-like cv. Désirée is not in the final viral concentration, but rather in the multiplication dynamics (**Figure 2A**). Although faster spread of *Plum pox virus* was observed in NahG tobacco plants [29], recently Wang [30] reported decreased viral concentration and alleviated symptom development in NahG *Arabidopsis* plants. Delayed appearance of disease symptoms after SA treatment was correlated with temporarily reduced replication of PVY at the early stage of infection, suggesting that the virus may have a mechanism to evade SA-mediated suppression of its replication and/or movement [27]. In upper non-inoculated leaves higher content of viral RNA was detected in cv. Désirée and NahG-Désirée than in cv. Igor; however its accumulation was not time-dependent (**Figure 2B, C**). It seems that after virus spreads to the upper leaves; its replication is fast, reaching its maximum concentration in some form of equilibrium. Thus systemic symptom development is not related to viral spread and accumulation in non-infected tissues, but to the initial specific plant response occurring in the inoculated leaves.

To investigate the time course of potato responses against PVY we have followed the expression of seven marker genes involved in defence against viruses, photosynthesis and carbohydrate metabolism. Positive correlations were observed within defence-related gene group and within photosynthesis/carbohydrate metabolism-related gene group, indicating their common molecular regulation. A clear distinction between the symptomatic and asymptomatic genotypes is visible on the level of the individual genotype and time-point profile from 5 dpi (**Figure S5**), proving that disease state indeed affects the metabolic status of the plant.

In most studies of plant-virus interaction reduction of the photosynthesis rate [31], [32], down-regulation of genes involved in photosynthesis [16], [20], [33], reduction of photosynthetic pigments [34] and structural changes of chloroplasts [35] have been associated with symptom expression. A gradual decrease in CAB and RuBisCO expression was observed during PVY^N^ infection of susceptible tobacco from 2 to 6 dpi [33]. Moreover, it was demonstrated that both virus infection and SA treatment of infected plants significantly decreased photosynthetic pigment content [36]. In our study the expression of photosynthesis-related genes discriminated the symptomatic from asymptomatic genotypes (**Figure S5**). The expression of photosynthesis-related genes was mostly correlated between each other (**Figure S1A, Figure S2A**), exhibiting similar dynamics (**Figure 3** **, Figure S1B, Figure S2B, Figure S3**). Interestingly, different time courses of the expression of GBSSI were observed in the inspected potato genotypes. When comparing the early response of cv. Igor inoculated with the aggressive PVY^NTN^ and the mild PVY^N^ isolates, plants infected with the PVY^N^ showed a higher expression of GBSSI, possibly leading to starch accumulation, and down-regulation of sucrose synthase, implying accumulation of soluble sugars [15]. These findings imply the importance of carbohydrate metabolism in plant-virus interaction, as has been demonstrated before [33], [37], [38].

Additionally, we have followed the expression levels of two groups of PR proteins, β-1,3-glucanases belonging to the PR-2 group of proteins and a member of the PR-1 protein family. The genes proved to be valuable markers of defence responses because their expression is induced and parallels the PVY RNA accumulation at the site of infection (**Figure 3** **, Figure S3**); similar to Glu-II expression in the *A. thaliana* and *Turnip mosaic virus* interaction [16]. However, in our study their induction preceded symptom development. Surprisingly, the response of PR protein genes was stronger in the symptomatic NahG-Désirée and cv. Igor genotypes than in cv. Désirée, indicating that PR gene expression is not directly related to the tolerance-like response observed in cv. Désirée.

Increased expression of all three classes of β-1,3-glucanase, a callose hydrolyzing enzyme, appears to promote the spread of viruses [23], [39], [40]. Within this study, β-1,3-glucanase genes expression was induced before the first detection of viral multiplication in all three genotypes, albeit the least in cv. Désirée (**Figure 3** **, Figure S3**). An opposite effect was reported by Linthorst and coworkers [41], where *Tobacco mosaic virus* (TMV) and SA strongly induced genes encoding acidic and basic β-1,3-glucanase in tobacco. Induction of β-1,3-glucanase genes was also observed in non-inoculated leaves (**Figure 4** **, Figure S4**). Although all β-1,3-glucanase genes showed induction over time, the expression profiles of different enzyme classes were different, both genotype- and site-specific (**Figure 3** **,** **Figure 4** **, Figure S5, Figure S3, Figure S4**). This result indicates diverse roles of different members of the enzyme family, as has already been reported in TMV infected tobacco [23]. Different profiles of expression of β-1,3-glucanase classes were also observed in the early response of sensitive potato cvs. Igor and Nadine to inoculation with two PVY isolates, where in general lower expression was detected in plants inoculated with the aggressive PVY^NTN^ than in plants inoculated with the mild PVY^N^ virus isolate [15]. Taken together this data, it is not possible to distinguish whether β-1,3-glucanases are induced in response to viral infection, as a consequence of activation of general defence mechanisms targeting various plant pathogens, or under viral control to enable faster spread of the virus.

Although their exact function is unknown, PR-1 proteins are often regarded as defence-related proteins [22]. Members of the PR-1 family are marker proteins of the SA-induced resistance response in many plant-pathogen interactions, but were also found to be important in compatible interactions with plant viruses (reviewed in Whitham [2]). Their expression was shown to be increased as the systemic symptoms progressed [27]. Navarre and Mayo [11] suggested that high basal SA levels observed in potato result in a high basal level of PR-1 expression. Similarly, the basal level of expression of PR-1b in cv. Désirée was 10-fold higher than in NahG-Désirée (0 dpi, **Table S4**). In our study the induction of PR-1b gene expression was stronger in symptomatic genotypes (**Figure 3** **, Figure S3**), which is in agreement with the results by Naderi and Berger [42]. Higher basal levels of SA and consequently of PR-1 in the cv. Désirée might be the reason for its weaker induction, but do not explain even more dramatic induction of PR-1 gene expression in cv. Igor. On the contrary, the response of defence related gene group in non-inoculated leaves of cv. Désirée was not very different from either of the symptomatic genotypes (**Figure 4** **, Figure S4**).

When comparing the kinetics of the expression of marker genes in both symptomatic cultivars, NahG-Désirée and Igor, it could be concluded that different molecular events can lead to similar disease outcomes (**Figure 3** **,** **Figure 4**). Significantly lower amounts of viral RNA in non-inoculated leaves (**Figure 2B, C**) might indicate that some sort of viral long distance movement arrest or multiplication restraint takes place in cv. Igor, which could be the consequence of high basal SA concentration. Moreover, the response of cv. Igor plants resembles an inefficient hypersensitive response (HR), observed in systemic necrosis development in the *Nicotiana benthamiana* and *Plantago asiatica mosaic virus* compatible interaction, where although HR and programmed cell death PCD occurred, they were not sufficient to stop the viral spread [**43**].

In conclusion, the importance of SA in viral multiplication and symptom development was confirmed through pronounced symptom development in NahG-Désirée and reversion of the effect after spraying with a SA analogue. We have shown that SA affects viral replication in the inoculated leaves, but does not prevent further spread of the virus. It is evident that the mechanisms of sensitivity, tolerance or resistance do not rely simply on one regulatory switch. Instead, a complex regulatory network is responsible for activation of a successful defence response. Consequently, similar morphological features (e.g. symptoms) can result from very different responses at the molecular level. In addition, it seems that not only the components involved but also the timing and intensity of response are extremely important for the outcome of a plant-virus interaction, be it disease, tolerance or resistance. Our results indicate that early molecular events at the site of the infection in fact influence further development of the disease. Thus, our study confirms the importance of dynamic studies of plant-virus interactions and may explain at least some inconsistencies in published data as due to differences in the timing of sampling that may be avoided if the time course of events is taken into account.

## Materials and Methods

### Plant material, inoculation and sampling

Three different potato genotypes (*Solanum tuberosum* L.) were used in the study: non-transgenic and NahG transgenic plants (lines NahG-D2 and NahG-A [19]) of cv. Désirée and plants of cv. Igor. The basal levels of selected genotypes are reported to be different: Désirée plants contain approximately 0.1 µg/g FW free SA, NahG-Désirée 0.01 µg/g FW [13] and cv. Igor 10 fold more than cv. Désirée, approximately 1 µg/g FW [12]. Healthy potato plants were grown in stem node tissue culture. Two weeks after node segmentation, they were transferred to soil in a growth chamber and kept at 21±2°C in the light and 18±1°C in the dark, at a relative humidity of 75%±2%, with 70–90 µmol/m^2^/s^2^ radiation (L36W/77 lamp, Osram, Germany) and a 16-h photoperiod. After four weeks of growth in soil, the potato plants were inoculated with PVY^NTN^ (isolate NIB-NTN, AJ585342) or mock inoculated as described in [14].

For analysis of dynamics of plant-virus interactions, plants of cv. Désirée, NahG-D2-Désirée line and cv. Igor were inoculated with PVY^NTN^. Leaves from three individual plants per genotype per time point were harvested; inoculated leaves at 3, 4, 5, 7 and 9 days after inoculation (dpi; at later times all inoculated leaves have fallen off) and non-inoculated leaves at 3, 4, 5, 7, 9, 10 and 11 dpi. To distinguish effects of viral inoculation from possible developmentally-regulated changes, we performed an additional independent experiment on cv. Désirée and NahG-Désirée. Leaves from three mock- and virus-inoculated plants were harvested before the experiment (time point 0) and at 3, 4, 5, and 7 dpi (inoculated leaves and non-inoculated leaves) as well as 8, 9 and 11 dpi (non-inoculated leaves). For study of viral amplification and spread, non-inoculated leaves of 8–10 plants of cv. Igor and the NahG-D2-Désirée line were harvested at 16 dpi.

For experiments with SA analogue treatment, NahG-Désirée (both lines) and cv. Igor were sprayed with 0.3 mM or 1 mM INA (98% 2,6-Dichloroisonicotinic acid, Aldrich, Dorset, UK) in distilled H_2_O or with distilled H_2_O alone (control) 24 hours before virus inoculation. The first bottom inoculated leaf and the first upper intact leaves from four individual plants were harvested at 6 and 14 dpi, respectively, for viral RNA accumulation measurements.

In all experiments, healthy and mock inoculated plants (inoculated with the sap of healthy plants as described above) were used as controls for symptom comparison.

### Quantitative real-time PCR

Relative concentration of PVY RNA [44] and expression of pathogenesis-related protein 1b (PR-1b), β-1,3-glucanase I (Glu I [45]), II (Glu II) and III (Glu III), RuBisCO activase (RA), chlorophyll a–b binding protein 4 (CAB4) and granule bound starch synthase I (GBSSI [15]) genes were followed using quantitative real-time PCR. Primers for PR-1b were designed as described in [14] and were 5′-GTATGAATAATTCCACGTACCATATGTTC-3′ (forward primer) and 5′-GTGGAAACAAGAAGATGCAATACTTAGT-3′ (reverse primer).

RNA was isolated from all samples using the RNeasy Plant Mini Kit (Qiagen, Hilden, Germany) according to the manufacturer's instructions, but with a modified elution step [14]. DNase-treated (Invitrogen, Carlsbad, CA; 0.1 U/DNase per µg RNA) total RNA (1–2 µg) was reverse transcribed using the High Capacity cDNA Reverse Transcription Kit (Applied Biosystems, Carlsbad, CA). Samples were analysed in the set-up for quantitative PCR analysis as previously described [46], using SYBR Green I chemistry for target host genes and TaqMan chemistry for viral RNA, cytochrome oxidase (Cox [47]) and 18S rRNA (Eukaryotic 18S rRNA TaqMan endogenous control, Applied Biosystems, Carlsbad, CA, USA). The standard curve method was used for relative gene expression quantification, and the transcript accumulation of each gene was normalized to Cox and 18S rRNA.

### Statistical analysis of viral RNA accumulation and host gene expression data

The results of gene expression analysis were calculated as relative transcript copy numbers scaled to the geometrical mean of both endogenous controls and to the average gene expression of leaf samples collected at the first time point (3 dpi) for the Désirée genotype. In the second independent experiment, data was normalized to average expression in the mock-inoculated samples.

Several types of statistical analysis were performed on the obtained dataset. Time courses of gene expression were visualised using Microsoft Excel. All further analyses and visualisations of results were performed in R statistical environment (R Development Core Team 2010). Data were log_2_ transformed and normalised using quantile normalisation to obtain comparative expression values for different genes. Correlation of gene expression profiles within the genotype was calculated. The similarity of samples within each genotype was presented using Euclidian distances. The following factors were included in analysis of variance: genotype, time after infection, viral accumulation at the site of inoculation, viral accumulation in non-inoculated leaves, primary symptom development, secondary symptom development and leaf yellowing.

## Supporting Information

Figure S1 **Potato gene expression and viral accumulation following PVY inoculation.** (A) Correlation between expression of different host genes and PVY RNA concentration. Correlation coefficients of host gene expression and viral accumulation in inoculated (left part) and upper non-inoculated leaves (right part) of three potato genotypes, cv. Désirée, NahG-Désirée and cv. Igor. PR-1b: pathogenesis-related protein 1b; Glu I, II, III: β-1,3-glucanase classes I, II, III; RA: RuBisCO activase; GBSSI: granule bound starch synthase I; CAB4: chlorophyll a–b binding protein 4; PVYi and PVYu: viral RNA in inoculated and upper leaves. Positive and negative values denote positive and negative correlations, respectively. Font size represents the relevance of the correlation (smallest font size denotes correlations smaller than 0.5). (B) Between-plant gene expression similarity in upper non-inoculated leaves. The colour of the small squares denotes similarity (Euclidian distance; the darker colour – the higher the similarity) between the overall expression profiles in individual plants of different potato genotypes (cv. Désirée, NahG-Désirée, cv. Igor), grouped by time point (3, 4, 5, 7, 9, 10, 11 dpi) after PVY inoculation.
(TIF)

Figure S2 **Potato gene expression and viral accumulation following PVY inoculation.** in the second independent experiment. (A) Correlation between expression of different host genes and PVY RNA concentration. Correlation coefficients of gene expression (PR-1b: pathogenesis-related protein 1b; Glu I, II, III: β-1,3-glucanase classes I, II, III; RA: RuBisCO activase; GBSSI: granule bound starch synthase I; CAB4: chlorophyll a–b binding protein 4) and viral accumulation (PVYi and PVYu: viral RNA in inoculated and upper leaves, respectively) in inoculated (lower left part) and upper non-inoculated (upper right part) leaves, normalized to expression in mock-inoculated samples of potato genotypes, cv. Désirée and NahG-Désirée. Positive and negative values denote positive and negative correlations, respectively. (B) Between-plant gene expression similarity in upper non-inoculated leaves. The colour of the small squares denotes similarity (Euclidian distance; darker colour higher similarity) between the overall expression profiles in individual plants (normalized to expression in mock-inoculated plants) of potato genotypes cv. Désirée and NahG-Désirée, grouped by time point (3, 4, 5, 7, 8, 9 and 11 dpi) after PVY inoculation. Outliers (second plant at 7 dpi and first plant at 11dpi in cv. Désirée and second plant at 9 and 11 dpi in NahG-Désirée) were excluded from further analyses.
(TIF)

Figure S3 **Dynamics of gene expression in inoculated leaves in the second independent experiment.** Expression of (A, C, E) defence-related marker genes (β-1,3-glucanase classes I, II, III: Glu-I, Glu-II, Glu-III; pathogenesis-related protein1b: PR-1b) and (B, D, F) photosynthesis (chlorophyll a-b binding protein 4: CAB4; and RuBisCO activase: RA) and carbohydrate metabolism (granule bound starch synthase I: GBSSI) marker genes, normalized to expression in mock-inoculated samples in inoculated leaves of cv. Désirée and NahG-Désirée at 3, 4, 5 and 7 days after infection (dpi). Relative viral RNA concentration (Pvy I) is plotted on each chart for easier comparison. Data points represent the mean of three measurements. Statistical evaluation of data is shown separately in **Table S3**.
(TIF)

Figure S4 **Dynamics of gene expression in upper non-inoculated leaves in the second experiment.** Expression of defence-related (β-1,3-glucanase of three classes: Glu-I, Glu-II, Glu-III; pathogenesis-related protein1b: PR-1b; A, C, E), photosynthesis (chlorophyll a–b binding protein 4: CAB4 and RuBisCO activase: RA) and carbohydrate metabolism (granule bound starch synthase I: GBSSI) marker genes (B, D, F), normalized to expression in mock-inoculated plants in upper non-inoculated leaves of potato genotypes cv. Désirée and NahG-Désirée at 3, 4, 5, 7, 8, 9 and 11 days after infection (dpi). Relative viral RNA concentration (Pvy I) in the inoculated leaves is plotted on each chart for easier comparison. Data points represent the mean of three measurements. Statistical evaluation of data is shown separately in **Table S3**.
(TIF)

Figure S5 **Gene expression and viral RNA accumulation profiles at 3, 4, 5, 7 and 9 days following PVY inoculation (dpi) in inoculated leaves of different potato genotypes (cv. Désirée, NahG-Désirée, cv. Igor).** Relative log_2_ expression values of each selected gene (PR-1b: pathogenesis-related protein 1b; Glu I, II, III: β-1,3-glucanase classes I, II, III; RA: RuBisCO activase; GBSSI: granule bound starch synthase I; CAB4: chlorophyll a-b binding protein 4) and PVY RNA (PVYi) in individual plants are represented with dots.
(TIF)

Table S1 **Symptom exhibition on potato plants of cv. Désirée, NahG-Désirée and cv. Igor, following PVY inoculation.** Percentages of inoculated leaves (n = 9; 3 leaves on 3 plants) showing local symptoms (necrosis, chlorosis), yellowing or having fallen off and the percentage of plants showing systemic symptoms from 3 to 11 days post inoculation (dpi) are shown.
(DOC)

Table S2 **Symptom development 5 to 28 days post PVY inoculation (dpi), after treatment of NahG-Désirée (lines NahG-A and NahG-D2) and cultivar Igor plants (n = 5) with distilled water (0), 0.3 mM or 1 mM INA 24 hours prior to viral inoculation.** Percentages of inoculated leaves showing local symptoms, yellowing or having fallen off and the percentage of plants showing systemic symptoms are shown. na – observed leaves had fallen off.
(XLS)

Table S3 **Significance of changes in expression of selected genes and viral RNA accumulation over time in different potato genotypes in the second independent experiment.**Significance of increase (+++: p<0.001, ++: p<0.01, +: p< 0.05, •: p<0.1) or decrease (---: p<0.001, --: p<0.01,-: p<0.05, •: p<0.1) in gene expression (PR-1b: pathogenesis-related protein 1b; Glu I, II, III: β-1,3-glucanase classes I, II, III; RA: RuBisCO activase; GBSSI: granule bound starch synthase I; CAB4: chlorophyll a–b binding protein 4) and viral accumulation (PvyI) is shown for comparisons between consecutive time points (upper panel) and to the first time point (0 dpi; middle panel), and comparison between virus and mock-inoculated samples (lower panel). An empty field denotes no significance.
(DOC)

Table S4 **Expression of selected host genes: raw data.** Relative gene expression of β-1,3-glucanase classes (Glu-I, Glu-II, Glu-III), pathogenesis-related protein1b (PR-1b), chlorophyll a–b binding protein 4 (CAB4), RuBisCO activase (RA) and granule bound starch synthase I (GBSSI) and relative viral RNA concentration (PVY), in inoculated and upper non-inoculated leaves of individual plants of potato genotypes cv. Désirée and NahG-Désirée at 3, 4, 5, 7, 8, 9 and 11 days after inoculation (dpi) with PVY or mock inoculation and in non-treated plants before start of experiment (0 dpi).
(PDF)
