# Supplementary figures and images for: Dynamics of Responses in Compatible Potato - Potato virus Y Interaction Are Modulated by Salicylic Acid
Source: PLoS One. 2011 Dec 14;6(12):e29009. doi: 10.1371/journal.pone.0029009 (PMC3237580; doi:10.1371/journal.pone.0029009)

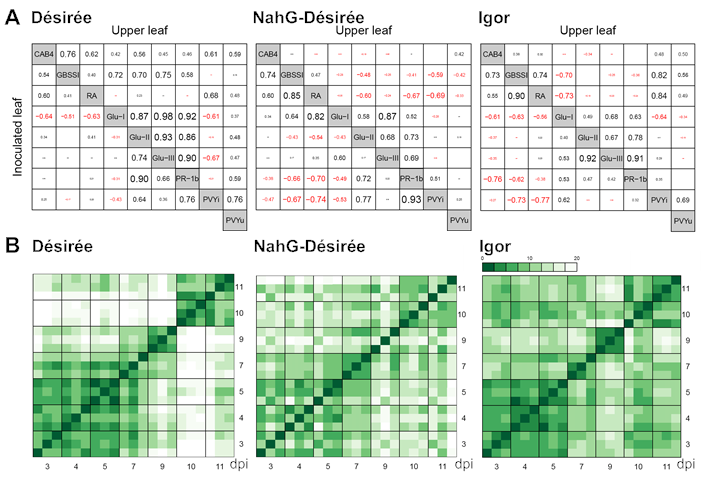

Supplement: Figure S1 — Potato gene expression and viral accumulation following PVY inoculation. (A) Correlation between expression of different host genes and PVY RNA concentration. Correlation coefficients of host gene expression and viral accumulation in inoculated (left part) and upper non-inoculated leaves (right part) of three potato genotypes, cv. Désirée, NahG-Désirée and cv. Igor. PR-1b: pathogenesis-related protein 1b; Glu I, II, III: β-1,3-glucanase classes I, II, III; RA: RuBisCO activase; GBSSI: granule bound starch synthase I; CAB4: chlorophyll a–b binding protein 4; PVYi and PVYu: viral RNA in inoculated and upper leaves. Positive and negative values denote positive and negative correlations, respectively. Font size represents the relevance of the correlation (smallest font size denotes correlations smaller than 0.5). (B) Between-plant gene expression similarity in upper non-inoculated leaves. The colour of the small squares denotes similarity (Euclidian distance; the darker colour – the higher the similarity) between the overall expression profiles in individual plants of different potato genotypes (cv. Désirée, NahG-Désirée, cv. Igor), grouped by time point (3, 4, 5, 7, 9, 10, 11 dpi) after PVY inoculation. (TIF) [file pone.0029009.s001.tif]

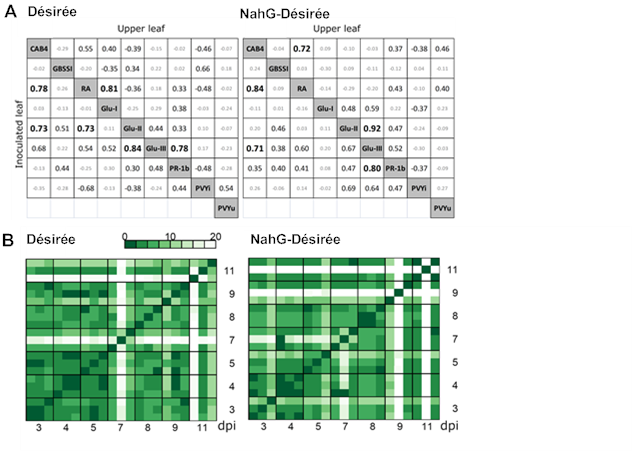

Supplement: Figure S2 — Potato gene expression and viral accumulation following PVY inoculation. in the second independent experiment. (A) Correlation between expression of different host genes and PVY RNA concentration. Correlation coefficients of gene expression (PR-1b: pathogenesis-related protein 1b; Glu I, II, III: β-1,3-glucanase classes I, II, III; RA: RuBisCO activase; GBSSI: granule bound starch synthase I; CAB4: chlorophyll a–b binding protein 4) and viral accumulation (PVYi and PVYu: viral RNA in inoculated and upper leaves, respectively) in inoculated (lower left part) and upper non-inoculated (upper right part) leaves, normalized to expression in mock-inoculated samples of potato genotypes, cv. Désirée and NahG-Désirée. Positive and negative values denote positive and negative correlations, respectively. (B) Between-plant gene expression similarity in upper non-inoculated leaves. The colour of the small squares denotes similarity (Euclidian distance; darker colour higher similarity) between the overall expression profiles in individual plants (normalized to expression in mock-inoculated plants) of potato genotypes cv. Désirée and NahG-Désirée, grouped by time point (3, 4, 5, 7, 8, 9 and 11 dpi) after PVY inoculation. Outliers (second plant at 7 dpi and first plant at 11dpi in cv. Désirée and second plant at 9 and 11 dpi in NahG-Désirée) were excluded from further analyses. (TIF) [file pone.0029009.s002.tif]

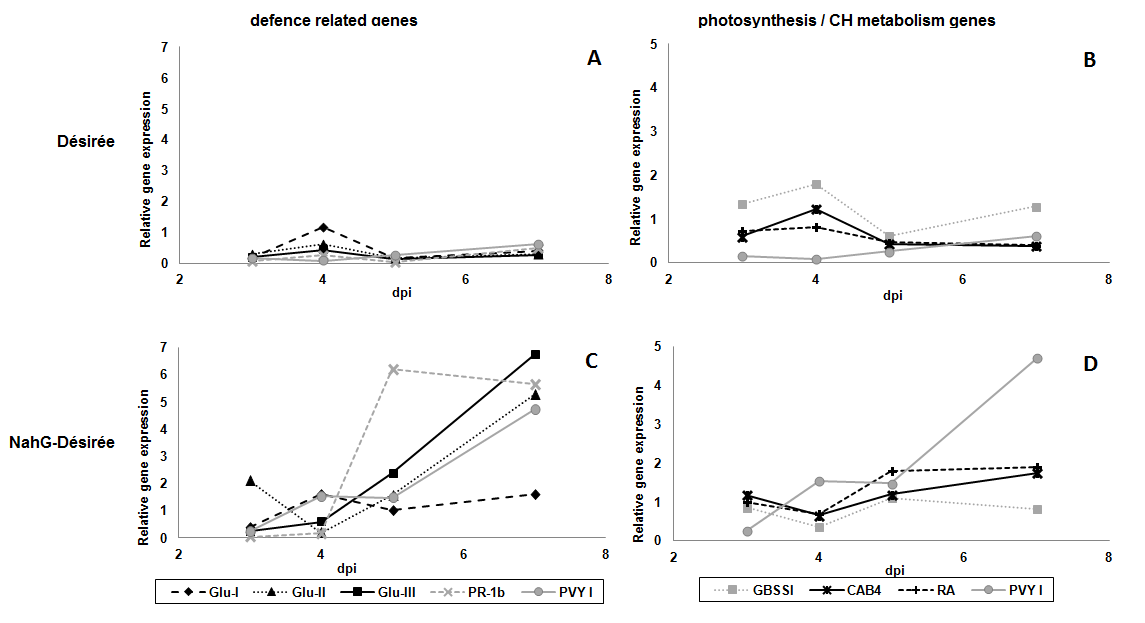

Supplement: Figure S3 — Dynamics of gene expression in inoculated leaves in the second independent experiment. Expression of (A, C, E) defence-related marker genes (β-1,3-glucanase classes I, II, III: Glu-I, Glu-II, Glu-III; pathogenesis-related protein1b: PR-1b) and (B, D, F) photosynthesis (chlorophyll a-b binding protein 4: CAB4; and RuBisCO activase: RA) and carbohydrate metabolism (granule bound starch synthase I: GBSSI) marker genes, normalized to expression in mock-inoculated samples in inoculated leaves of cv. Désirée and NahG-Désirée at 3, 4, 5 and 7 days after infection (dpi). Relative viral RNA concentration (Pvy I) is plotted on each chart for easier comparison. Data points represent the mean of three measurements. Statistical evaluation of data is shown separately in Table S3. (TIF) [file pone.0029009.s003.tif]

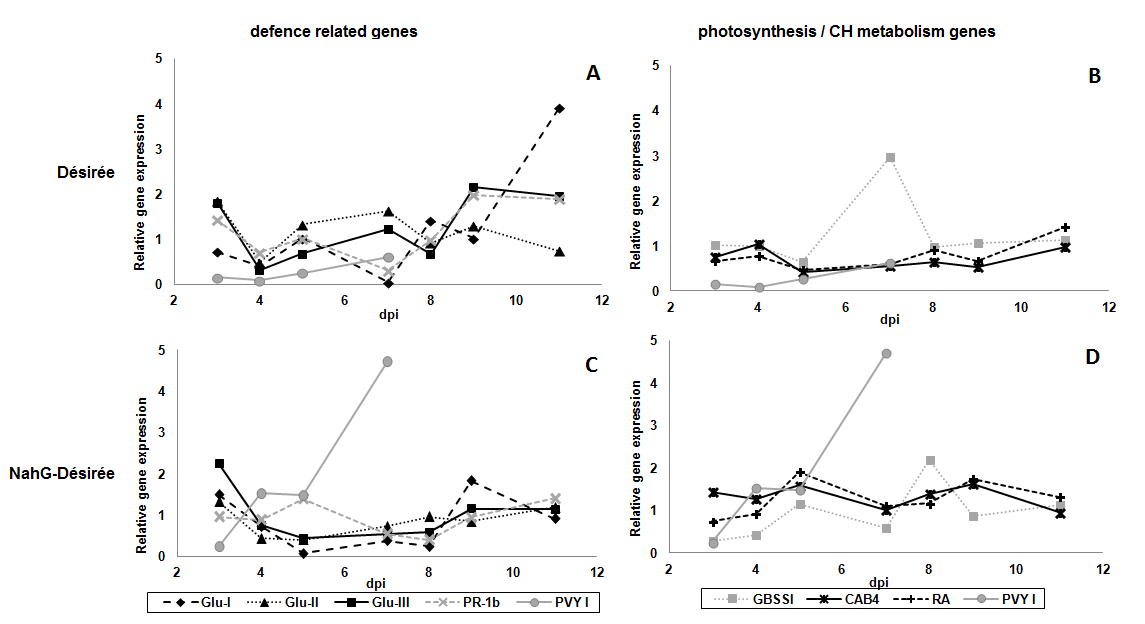

Supplement: Figure S4 — Dynamics of gene expression in upper non-inoculated leaves in the second experiment. Expression of defence-related (β-1,3-glucanase of three classes: Glu-I, Glu-II, Glu-III; pathogenesis-related protein1b: PR-1b; A, C, E), photosynthesis (chlorophyll a–b binding protein 4: CAB4 and RuBisCO activase: RA) and carbohydrate metabolism (granule bound starch synthase I: GBSSI) marker genes (B, D, F), normalized to expression in mock-inoculated plants in upper non-inoculated leaves of potato genotypes cv. Désirée and NahG-Désirée at 3, 4, 5, 7, 8, 9 and 11 days after infection (dpi). Relative viral RNA concentration (Pvy I) in the inoculated leaves is plotted on each chart for easier comparison. Data points represent the mean of three measurements. Statistical evaluation of data is shown separately in Table S3. (TIF) [file pone.0029009.s004.tif]

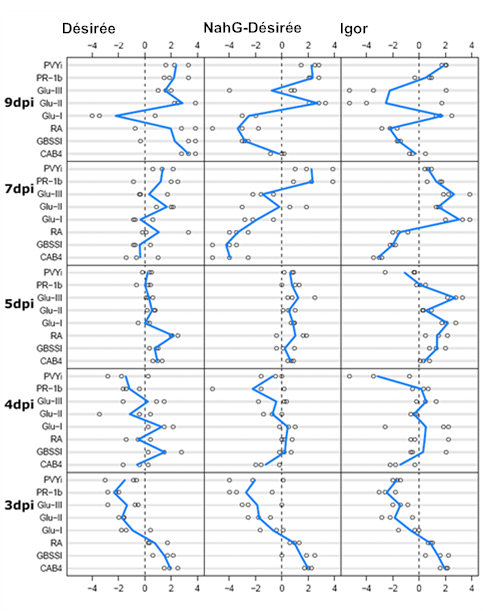

Supplement: Figure S5 — Gene expression and viral RNA accumulation profiles at 3, 4, 5, 7 and 9 days following PVY inoculation (dpi) in inoculated leaves of different potato genotypes (cv. Désirée, NahG-Désirée, cv. Igor). Relative log2 expression values of each selected gene (PR-1b: pathogenesis-related protein 1b; Glu I, II, III: β-1,3-glucanase classes I, II, III; RA: RuBisCO activase; GBSSI: granule bound starch synthase I; CAB4: chlorophyll a-b binding protein 4) and PVY RNA (PVYi) in individual plants are represented with dots. (TIF) [file pone.0029009.s005.tif]
