# Supplementary material for: Dynamics of Responses in Compatible Potato - Potato virus Y Interaction Are Modulated by Salicylic Acid
Source: PLoS One. 2011 Dec 14;6(12):e29009. doi: 10.1371/journal.pone.0029009 (PMC3237580; doi:10.1371/journal.pone.0029009)
Supplement: Table S1 — Symptom exhibition on potato plants of cv. Désirée, NahG-Désirée and cv. Igor, following PVY inoculation. Percentages of inoculated leaves (n = 9; 3 leaves on 3 plants) showing local symptoms (necrosis, chlorosis), yellowing or having fallen off and the percentage of plants showing systemic symptoms from 3 to 11 days post inoculation (dpi) are shown. (DOC) [file pone.0029009.s006.doc]

| Genotype | dpi | Disease symptoms | | | |
| --- | --- | --- | --- | --- | --- |
|  |  | Local symptoms | Local yellowing | Fallen off | Systemic |
| Désirée | 3 | 0 | 0 | 0 | 0 |
|  | 4 | 0 | 0 | 0 | 0 |
|  | 5 | 0 | 78 | 0 | 0 |
|  | 7 | 0 | 100 | 0 | 0 |
|  | 9 | 0 | 100 | 0 | 0 |
|  | 10 | 0 | 100 | 0 | 0 |
|  | 11 | 0 | 100 | 0 | 0 |
| NahG- Désirée | 3 | 0 | 0 | 0 | 0 |
|  | 4 | 0 | 0 | 0 | 0 |
|  | 5 | 67 | 67 | 0 | 0 |
|  | 7 | 89 | 89 | 0 | 0 |
|  | 9 | 89 | 89 | 22 | 0 |
|  | 10 | 100 | 100 | 33 | 2 |
|  | 11 | 100 | 100 | 33 | 67 |
| Igor | 3 | 0 | 0 | 0 | 0 |
|  | 4 | 0 | 0 | 0 | 0 |
|  | 5 | 67 | 67 | 0 | 0 |
|  | 7 | 100 | 100 | 0 | 0 |
|  | 9 | 100 | 100 | 33 | 0 |
|  | 10 | 100 | 100 | 66 | 0 |
|  | 11 | 100 | 100 | 100 | 33 |
