# Supplementary material for: Dynamics of Responses in Compatible Potato - Potato virus Y Interaction Are Modulated by Salicylic Acid
Source: PLoS One. 2011 Dec 14;6(12):e29009. doi: 10.1371/journal.pone.0029009 (PMC3237580; doi:10.1371/journal.pone.0029009)
Supplement: Table S3 — Significance of changes in expression of selected genes and viral RNA accumulation over time in different potato genotypes in the second independent experiment.Significance of increase (+++: p<0.001, ++: p<0.01, +: p< 0.05, •: p<0.1) or decrease (---: p<0.001, --: p<0.01,-: p<0.05, •: p<0.1) in gene expression (PR-1b: pathogenesis-related protein 1b; Glu I, II, III: β-1,3-glucanase classes I, II, III; RA: RuBisCO activase; GBSSI: granule bound starch synthase I; CAB4: chlorophyll a–b binding protein 4) and viral accumulation (PvyI) is shown for comparisons between consecutive time points (upper panel) and to the first time point (0 dpi; middle panel), and comparison between virus and mock-inoculated samples (lower panel). An empty field denotes no significance. (DOC) [file pone.0029009.s008.doc]

| **a)** | | **Inoculated leaves** | | | | | | | | | | | | | | | | | | | | | | | |  | | | | | | | **Upper non−inoculated leaves** | | | | | | | | | | | | | | | | | |  | |
| --- | --- | --- | --- | --- | --- | --- | --- | --- | --- | --- | --- | --- | --- | --- | --- | --- | --- | --- | --- | --- | --- | --- | --- | --- | --- | --- | --- | --- | --- | --- | --- | --- | --- | --- | --- | --- | --- | --- | --- | --- | --- | --- | --- | --- | --- | --- | --- | --- | --- | --- | --- | --- |
|  | | |  | |  | |  | | |  | | |  | |  | |  | |  | |  |  | | | | | | |  | | |  | | | |  | | |  | | |  | | |  | | |  | | | |  |
|  | | |  | | Glu-I | | Glu-II | | | Glu-III | | | PR-1b | | CAB4 | | RA | | GBSSI | |  |  | | | | | | | Glu-I | | | Glu-II | | | | Glu-III | | | PR-1b | | | CAB4 | | | RA | | | GBSSI | | | |  |
| Désirée | | | 3 dpi | |  | |  | |  | | |  | | |  | | −− | |  | |  | | | 3 dpi | | | | ++ | | | + | | | |  | | | • | | |  | | | − | | | • | | |  | | |
|  |  |  | 4 dpi | |  | |  | |  | | |  | | |  | |  | |  | |  | | | 4 dpi | | | |  | | |  | | | |  | | |  | | |  | | |  | | |  | | |  | | |
|  |  |  | 5 dpi | |  | |  | |  | | |  | | | − | | − | |  | |  | | | | 5 dpi | | |  | | |  | | | |  | | |  | | |  | | | • | | |  | | |  | | |
|  |  |  | 7 dpi | |  | |  | |  | | |  | | |  | |  | |  | |  | | | | 7 dpi | | | − | | | + | | | |  | | |  | | | − | | | −− | | | • | | |  | | |
|  |  |  |  | |  | |  | |  | | |  | | |  | |  | |  | |  | | | | 8 dpi | | |  | | |  | | | |  | | |  | | | − | | |  | | |  | | |  | | |
|  |  |  |  | |  | |  | |  | | |  | | |  | |  | |  | |  | | | | 9 dpi | | | ++ | | |  | | | |  | | |  | | | − | | | • | | |  | | |  | | |
|  |  |  |  |  |  |  |  |  |  |  |  |  |  |  |  |  |  |  |  |  |  |  |  |  | 11 dpi | | |  | | |  | | | | + | | |  | | |  | | |  | | |  | | |  | | |
| NahG- Désirée | | | 3 dpi | |  | | + | |  | | |  | | |  | |  | |  | |  | | | | 3 dpi | | | • | | |  | | | |  | | |  | | | + | | |  | | | −− | | |  | | |
|  |  |  | 4 dpi | |  | |  | |  | | |  | | |  | | − | | −− | |  | | | | 4 dpi | | |  | | |  | | | |  | | |  | | | • | | |  | | |  | | |  | | |
|  |  |  | 5 dpi | |  | |  | | + | | | + | | |  | |  | |  | |  | | | | 5 dpi | | |  | | |  | | | |  | | |  | | |  | | |  | | |  | | |  | | |
|  |  |  | 7 dpi | | + | | + | | + | | | + | | |  | |  | |  | |  | | | | 7 dpi | | |  | | |  | | | |  | | |  | | |  | | |  | | |  | | |  | | |
|  |  |  |  | |  | |  | |  | | |  | | |  | |  | |  | |  | | | | 8 dpi | | |  | | |  | | | |  | | |  | | | + | | |  | | | + | | |  | | |
|  |  |  |  | |  | |  | |  | | |  | | |  | |  | |  | |  | | | | 9 dpi | | | + | | |  | | | |  | | |  | | |  | | |  | | |  | | |  | | |
|  |  |  |  |  |  |  |  |  |  |  |  |  |  |  |  |  |  |  |  |  |  |  |  |  | 11 dpi | | | + | | |  | | | | • | | | ++ | | |  | | |  | | |  | | |  | | |
|  | **b)** | | |  | |  | |  | | |  | | |  | |  | |  | |  | | |  | | | |  | | |  | | | |  | | |  | | |  | | |  | | |  | | |  | | | |
|  |  | | |  | | Glu-I | | Glu-II | | | Glu-III | | | PR-1b | | CAB4 | | RA | | GBSSI | | | PvyI | | | |  | | | Glu-I | | | | Glu-II | | | Glu-III | | | PR-1b | | | CAB4 | | | RA | | | GBSSI | | | |
|  | Désirée | | | dpi 4:3 | | + | |  | | | + | | | + | | • | |  | |  | | |  | | | | dpi 4:3 | | |  | | | | −− | | |  | | |  | | | + | | |  | | |  | | | |
|  |  |  |  | dpi 5:4 | | − | | − | | | −− | | | − | | • | |  | |  | | |  | | | | dpi 5:4 | | |  | | | |  | | |  | | |  | | | −−− | | |  | | |  | | | |
|  |  |  |  | dpi 7:5 | |  | |  | | |  | | | ++ | |  | |  | |  | | | + | | | | dpi 7:5 | | |  | | | |  | | |  | | |  | | |  | | |  | | | +++ | | | |
|  |  |  |  |  | |  | |  | | |  | | |  | |  | |  | |  | | |  | | | | dpi 8:7 | | |  | | | |  | | |  | | |  | | |  | | |  | | | −−− | | | |
|  |  |  |  |  | |  | |  | | |  | | |  | |  | |  | |  | | |  | | | | dpi 9:8 | | |  | | | |  | | | • | | |  | | |  | | |  | | |  | | | |
|  |  |  |  |  |  |  |  |  |  |  |  |  |  |  |  |  |  |  |  |  |  |  |  |  |  |  | dpi 11:9 | | | + | | | |  | | |  | | |  | | | ++ | | | +++ | | |  | | | |
|  |  |  |  | dpi 3:0 | |  | |  | | |  | | |  | |  | | − | |  | | |  | | | | dpi 3:0 | | |  | | | | ++ | | |  | | |  | | |  | | | −− | | |  | | | |
|  |  |  |  | dpi 4:0 | | + | |  | | | • | | |  | |  | | • | |  | | |  | | | | dpi 4:0 | | |  | | | |  | | |  | | |  | | |  | | | − | | |  | | | |
|  |  |  |  | dpi 5:0 | |  | |  | | |  | | |  | |  | | − | |  | | |  | | | | dpi 5:0 | | |  | | | |  | | |  | | |  | | | −− | | | −− | | |  | | | |
|  |  |  |  | dpi 7:0 | |  | |  | | |  | | | + | |  | | − | |  | | | ++ | | | | dpi 7:0 | | |  | | | |  | | |  | | |  | | | − | | | −− | | | +++ | | | |
|  |  |  |  |  | |  | |  | | |  | | |  | |  | |  | |  | | |  | | | | dpi 8:0 | | |  | | | |  | | |  | | |  | | | • | | | • | | |  | | | |
|  |  |  |  |  | |  | |  | | |  | | |  | |  | |  | |  | | |  | | | | dpi 9:0 | | |  | | | |  | | | • | | |  | | | − | | | −− | | |  | | | |
|  |  |  |  |  |  |  |  |  |  |  |  |  |  |  |  |  |  |  |  |  |  |  |  |  |  |  | dpi 11:0 1111111111:0 | | | ++ | | | |  | | |  | | |  | | |  | | | + | | |  | | | |
|  | NahG- Désirée | | | dpi 4:3 | |  | |  | | |  | | |  | |  | |  | |  | | |  | | | | dpi 4:3 | | |  | | | |  | | |  | | |  | | |  | | |  | | |  | | | |
|  |  |  |  | dpi 5:4 | |  | |  | | |  | | | ++ | |  | | • | |  | | |  | | | | dpi 5:4 | | |  | | | |  | | |  | | |  | | | • | | | ++ | | | • | | | |
|  |  |  |  | dpi 7:5 | |  | | +++ | | | + | | |  | |  | |  | |  | | | +++ | | | | dpi 7:5 | | |  | | | |  | | |  | | |  | | | − | | | −− | | |  | | | |
|  |  |  |  |  | |  | |  | | |  | | |  | |  | |  | |  | | |  | | | | dpi 8:7 | | |  | | | |  | | |  | | |  | | |  | | |  | | | +++ | | | |
|  |  |  |  |  | |  | |  | | |  | | |  | |  | |  | |  | | |  | | | | dpi 9:8 | | | + | | | |  | | |  | | |  | | |  | | | + | | | −−− | | | |
|  |  |  |  |  |  |  |  |  |  |  |  |  |  |  |  |  |  |  |  |  |  |  |  |  |  |  | dpi 11:9 9:81111:9 | | |  | | | |  | | |  | | |  | | | − | | | • | | |  | | | |
|  |  |  |  | dpi 3:0 | |  | |  | | |  | | |  | |  | |  | |  | | |  | | | | dpi 3:0 | | | + | | | |  | | |  | | |  | | |  | | |  | | |  | | | |
|  |  |  |  | dpi 4:0 | |  | |  | | |  | | |  | |  | |  | |  | | |  | | | | dpi 4:0 | | |  | | | |  | | |  | | |  | | |  | | |  | | |  | | | |
|  |  |  |  | dpi 5:0 | |  | |  | | | • | | | ++ | |  | |  | |  | | |  | | | | dpi 5:0 | | |  | | | |  | | |  | | |  | | | + | | | ++ | | |  | | | |
|  |  |  |  | dpi 7:0 | |  | | +++ | | | +++ | | | + | |  | |  | |  | | | +++ | | | | dpi 7:0 | | |  | | | |  | | |  | | |  | | |  | | |  | | |  | | | |
|  |  |  |  |  | |  | |  | | |  | | |  | |  | |  | |  | | |  | | | | dpi 8:0 | | |  | | | |  | | |  | | |  | | |  | | |  | | | +++ | | | |
|  |  |  |  |  | |  | |  | | |  | | |  | |  | |  | |  | | |  | | | | dpi 9:0 | | | + | | | |  | | |  | | |  | | | + | | | + | | |  | | | |
|  |  |  |  |  |  |  |  |  |  |  |  |  |  |  |  |  |  |  |  |  | | |  | | | | dpi 11:0 11119:0dpi11:0 | | |  | | | |  | | |  | | |  | | |  | | |  | | | | | | |

**Supplemental table S8**. Significance of changes in expression of selected genes and viral RNA accumulation different potato genotypes in the second independent experiment.

Significances of increase (+++: p<0.001, ++: p<0.01, +: p< 0.05, •: p<0.1) or decrease (---: p<0.001, --: p<0.01,-: p< 0.05, •: p<0.1) in gene expression (PR-1b: pathogenesis-related protein 1b; Glu I, II, III: β-1,3-glucanase classes I, II, III; RA: RuBisCO activase; GBSSI: granule bound starch synthase I; CAB4: chlorophyll a-b binding protein 4) and viral accumulation (PvyI) are shown for comparisons between virus and mock-inoculated samples (a), between consecutive time points, and to the first time point (0 dpi; b). An empty field denotes no significance.
