# Supplementary material for: Dynamics of Responses in Compatible Potato - Potato virus Y Interaction Are Modulated by Salicylic Acid
Source: PLoS One. 2011 Dec 14;6(12):e29009. doi: 10.1371/journal.pone.0029009 (PMC3237580; doi:10.1371/journal.pone.0029009)
Supplement: Table S4 — Expression of selected host genes: raw data. Relative gene expression of β-1,3-glucanase classes (Glu-I, Glu-II, Glu-III), pathogenesis-related protein1b (PR-1b), chlorophyll a–b binding protein 4 (CAB4), RuBisCO activase (RA) and granule bound starch synthase I (GBSSI) and relative viral RNA concentration (PVY), in inoculated and upper non-inoculated leaves of individual plants of potato genotypes cv. Désirée and NahG-Désirée at 3, 4, 5, 7, 8, 9 and 11 days after inoculation (dpi) with PVY or mock inoculation and in non-treated plants before start of experiment (0 dpi). (PDF) [file pone.0029009.s009.pdf]

Supplemental table S9. Dynamics of gene expression: raw data

Relative gene expression of β-1,3-glucanase classes (Glu-I, Glu-II, Glu-III), pathogenesis-related protein1b (PR-1b), chlorophyll a-b binding protein 4 (CAB4), RuBisCO activase (RA) and granule bound starch synthase I (GBSSI) and relative viral RNA concentration (PVY), in inoculated and upper non-inoculated leaves of individual plants of potato genotypes Désirée and NahG-Désirée-Désirée at 3, 4, 5, 7, 8, 9 and 11 days after inoculation (dpi) with PVY or mock inoculation and in non-treated plants before start of experiment (0 dpi).

Genotype	Inoculum	Leaf type	dpi	PVY	PR-1b	Glu-I	Glu-II	Glu-II	RA	CAB4	GBSSI
Désirée		upper	0	0.00	1.00	1.00	1.00	1.00	1.00	1.00	1.00
Désirée		upper	0	0.00	0.26	1.52	0.32	0.33	1.09	0.72	0.73
Désirée		upper	0	0.00	0.44	0.43	0.81	0.67	1.39	1.68	2.24
Désirée		upper	0	0.00	0.05	NA	0.30	0.13	1.66	1.97	2.76
Désirée		upper	0	0.00	0.12	1.21	0.53	0.58	1.72	2.14	2.83
Désirée		upper	0	0.00	0.04	0.11	0.14	0.06	1.65	2.21	1.97
Désirée	MOCK	inoculated	1	0.00	NA	1.05	1.34	1.60	0.68	0.29	1.65
Désirée	MOCK	inoculated	1	0.00	0.69	0.27	0.76	0.55	1.03	0.59	1.77
Désirée	MOCK	inoculated	1	0.00	0.11	0.29	0.19	0.19	0.64	0.78	0.48
Désirée	MOCK	inoculated	3	0.00	2.11	0.82	0.84	1.10	0.82	0.82	1.22
Désirée	MOCK	inoculated	3	0.00	0.29	0.38	0.17	0.19	0.81	0.63	0.97
Désirée	MOCK	inoculated	3	0.00	1.76	0.97	1.51	1.45	0.83	0.99	1.04
Désirée	MOCK	inoculated	4	0.00	0.09	0.06	0.38	0.19	0.66	0.81	1.35
Désirée	MOCK	inoculated	4	0.00	0.02	0.02	0.25	0.06	1.23	1.58	1.43
Désirée	MOCK	inoculated	4	0.00	0.04	0.04	0.16	0.04	0.83	0.91	0.90
Désirée	MOCK	inoculated	5	0.00	0.55	0.27	0.86	0.35	1.13	0.93	1.60
Désirée	MOCK	inoculated	5	0.00	0.04	0.01	0.40	0.10	0.69	0.81	3.86
Désirée	MOCK	inoculated	5	0.00	1.38	0.33	1.55	0.66	0.89	0.88	1.04
Désirée	MOCK	inoculated	7	0.00	0.09	0.67	0.19	0.14	0.41	0.33	0.53
Désirée	MOCK	inoculated	7	0.00	0.55	0.11	0.36	0.40	0.87	0.51	0.77
Désirée	MOCK	inoculated	7	0.00	0.88	0.10	3.94	1.74	1.68	2.27	3.32
Désirée	MOCK	upper	1	0.00	0.04	0.02	0.46	0.19	2.37	3.28	3.38
Désirée	MOCK	upper	1	0.00	0.06	0.02	0.23	0.09	2.32	3.15	3.10
Désirée	MOCK	upper	1	0.00	0.02	0.02	0.05	0.02	1.75	2.10	2.36
Désirée	MOCK	upper	3	0.00	0.02	0.04	0.21	0.03	1.94	2.09	2.96
Désirée	MOCK	upper	3	0.00	0.04	0.04	0.15	0.03	1.39	1.58	1.88
Désirée	MOCK	upper	3	0.00	0.04	0.04	0.12	0.03	1.80	2.20	2.65
Désirée	MOCK	upper	4	0.00	0.16	0.03	1.39	0.69	1.23	1.36	5.40
Désirée	MOCK	upper	4	0.00	0.03	0.27	0.13	0.12	0.36	0.51	0.28
Désirée	MOCK	upper	4	0.00	0.03	0.02	0.13	0.03	1.74	1.68	2.31
Désirée	MOCK	upper	5	0.00	0.16	0.06	0.25	0.49	1.87	2.30	3.77
Désirée	MOCK	upper	5	0.00	0.15	0.04	0.69	0.18	0.81	0.96	3.12
Désirée	MOCK	upper	5	0.00	0.06	0.03	0.43	0.07	1.17	0.88	3.18
Désirée	MOCK	upper	7	0.00	0.29	0.08	0.18	0.07	0.92	1.06	0.62
Désirée	MOCK	upper	7	0.00	0.17	0.42	0.23	0.11	0.78	0.88	0.59
Désirée	MOCK	upper	7	0.00	0.05	0.06	0.28	0.06	0.97	1.23	1.03
Désirée	MOCK	upper	8	0.00	0.62	0.10	1.40	0.80	0.58	0.74	3.38
Désirée	MOCK	upper	8	0.00	0.70	0.24	0.86	0.45	0.54	0.58	6.06
Désirée	MOCK	upper	8	0.00	0.15	0.24	0.36	0.18	0.47	0.77	3.67
Désirée	MOCK	upper	9	0.00	0.06	0.05	0.59	0.16	0.69	0.81	5.21
Désirée	MOCK	upper	9	0.00	2.38	0.15	2.96	1.86	0.55	0.66	5.10
Désirée	MOCK	upper	9	0.00	1.02	0.18	0.89	0.34	0.40	0.54	4.19
Désirée	MOCK	upper	11	0.00	0.54	0.01	1.11	0.28	0.56	0.72	4.85

Genotype	Inoculum	Leaf type	dpi	PVY	PR-1b	Glu-I	Glu-II	Glu-II	RA	CAB4	GBSSI
Désirée	MOCK	upper	11	0.00	0.74	0.03	1.49	0.41	0.40	0.70	2.87
Désirée	MOCK	upper	11	0.00	0.82	0.03	1.78	0.52	0.49	0.58	4.03
Désirée	PVY	inoculated	1	1.00	0.56	1.30	0.41	0.35	0.37	0.57	1.26
Désirée	PVY	inoculated	1	3.75	0.33	0.91	0.45	0.34	0.76	0.85	2.40
Désirée	PVY	inoculated	1	0.77	0.75	0.90	0.65	0.37	0.52	0.77	1.73
Désirée	PVY	inoculated	3	0.39	0.18	0.83	0.34	0.63	0.41	0.40	0.86
Désirée	PVY	inoculated	3	0.20	1.84	0.64	1.21	1.06	0.58	0.73	2.21
Désirée	PVY	inoculated	3	0.42	0.24	0.38	0.17	0.16	0.38	0.33	1.04
Désirée	PVY	inoculated	4	0.11	0.12	0.07	0.34	0.13	0.47	1.16	3.25
Désirée	PVY	inoculated	4	0.29	0.12	0.44	0.19	0.14	0.42	0.72	1.63
Désirée	PVY	inoculated	4	0.18	0.03	0.13	0.61	0.17	0.82	2.19	1.37
Désirée	PVY	inoculated	5	0.96	0.05	0.30	0.17	0.06	0.13	0.13	0.15
Désirée	PVY	inoculated	5	0.26	0.33	0.17	0.69	0.33	0.34	0.39	1.44
Désirée	PVY	inoculated	5	0.43	0.24	0.03	0.31	0.18	0.49	0.59	2.16
Désirée	PVY	inoculated	7	1.21	1.28	0.14	1.32	0.80	0.41	0.73	2.88
Désirée	PVY	inoculated	7	1.50	3.02	1.36	1.24	1.06	0.18	0.20	0.99
Désirée	PVY	inoculated	7	1.29	0.97	0.21	0.50	0.34	0.29	0.26	1.72
Désirée	PVY	upper	1	0.00	0.05	0.13	0.41	0.58	2.05	2.59	8.22
Désirée	PVY	upper	1	0.00	0.03	0.02	0.10	0.02	1.11	1.62	3.71
Désirée	PVY	upper	1	0.00	0.06	0.03	0.14	0.01	1.17	2.20	5.14
Désirée	PVY	upper	3	0.00	0.06	0.13	0.43	0.08	0.87	1.72	2.92
Désirée	PVY	upper	3	0.00	0.05	0.12	0.38	0.06	1.05	1.58	3.54
Désirée	PVY	upper	3	0.00	0.09	0.18	0.30	0.19	1.00	1.40	3.76
Désirée	PVY	upper	4	0.00	0.12	0.32	0.70	0.38	0.77	1.33	6.33
Désirée	PVY	upper	4	0.00	0.05	0.21	0.20	0.06	0.78	1.51	2.54
Désirée	PVY	upper	4	0.00	0.04	0.11	0.07	0.05	0.63	1.14	1.82
Désirée	PVY	upper	5	0.00	0.10	0.52	0.54	0.28	0.35	NA	0.57
Désirée	PVY	upper	5	0.00	0.33	0.10	1.05	0.47	0.55	0.65	5.63
Désirée	PVY	upper	5	0.00	0.12	0.06	0.65	0.17	0.60	0.59	2.54
Désirée	PVY	upper	7	0.01	0.07	0.04	0.43	0.10	0.49	0.70	2.34
Désirée	PVY	upper	7	0.00	0.29	0.04	0.92	0.32	0.40	0.62	12.83
Désirée	PVY	upper	7	0.00	0.08	0.04	0.49	0.25	0.40	0.55	3.65
Désirée	PVY	upper	8	0.00	1.46	0.12	1.50	0.67	0.31	0.38	9.62
Désirée	PVY	upper	8	0.01	0.23	0.32	0.70	0.33	0.42	0.56	4.63
Désirée	PVY	upper	8	0.00	0.35	3.74	0.75	0.77	0.50	0.52	3.09
Désirée	PVY	upper	9	0.00	6.52	0.71	3.83	5.42	0.26	0.37	9.96
Désirée	PVY	upper	9	0.00	0.54	0.83	0.79	0.72	0.40	0.42	2.88
Désirée	PVY	upper	9	0.00	2.67	0.44	2.41	3.20	0.26	0.38	7.98
Désirée	PVY	upper	11	0.00	1.14	0.87	0.80	0.78	0.89	0.91	3.29
Désirée	PVY	upper	11	0.00	1.15	0.18	1.40	1.22	0.48	0.61	7.77
Désirée	PVY	upper	11	0.00	2.63	0.69	1.26	1.66	0.69	0.81	4.19
NahG-Désirée		upper	0	0.00	0.03	0.17	0.27	0.08	0.85	0.82	3.90
NahG-Désirée		upper	0	0.00	0.02	0.05	0.10	0.03	0.93	0.92	2.30
NahG-Désirée		upper	0	0.00	0.05	0.18	0.11	0.04	0.58	0.73	2.34
NahG-Désirée		upper	0	0.00	0.03	0.00	0.05	0.01	0.87	1.00	3.01
NahG-Désirée		upper	0	0.00	0.02	0.00	0.01	0.01	1.58	1.59	4.81
NahG-Désirée		upper	0	0.00	0.05	0.02	0.08	0.02	1.45	1.82	4.30
NahG-Désirée	MOCK	inoculated	1	0.00	0.12	0.15	0.05	0.06	0.82	0.52	2.52
NahG-Désirée	MOCK	inoculated	1	0.00	0.02	0.07	0.05	0.03	0.51	0.30	0.62
NahG-Désirée	MOCK	inoculated	1	0.00	0.32	0.07	0.12	0.08	0.99	0.58	3.40

Genotype	Inoculum	Leaf type	dpi	PVY	PR-1b	Glu-I	Glu-II	Glu-II	RA	CAB4	GBSSI
NahG-Désirée	MOCK	inoculated	3	0.00	0.08	0.08	0.08	0.08	0.66	0.37	1.28
NahG-Désirée	MOCK	inoculated	3	0.00	0.66	0.24	0.06	0.20	0.63	0.32	0.99
NahG-Désirée	MOCK	inoculated	3	0.00	1.08	0.04	0.23	0.21	0.53	0.21	3.30
NahG-Désirée	MOCK	inoculated	4	0.00	0.57	0.08	0.27	0.35	0.83	0.92	2.77
NahG-Désirée	MOCK	inoculated	4	0.00	1.04	0.13	0.94	0.60	0.59	0.50	1.96
NahG-Désirée	MOCK	inoculated	4	0.00	0.11	NA	0.27	0.12	0.56	0.88	2.59
NahG-Désirée	MOCK	inoculated	5	0.00	0.48	0.15	0.38	0.59	0.37	0.37	3.41
NahG-Désirée	MOCK	inoculated	5	0.00	0.10	0.28	0.17	0.18	0.31	0.46	0.55
NahG-Désirée	MOCK	inoculated	5	0.00	0.02	0.11	0.14	0.03	0.38	0.56	0.76
NahG-Désirée	MOCK	inoculated	7	0.00	0.05	0.48	0.19	0.08	0.18	0.09	1.14
NahG-Désirée	MOCK	inoculated	7	0.00	0.08	1.29	0.33	0.08	0.20	0.10	0.42
NahG-Désirée	MOCK	inoculated	7	0.00	0.60	0.06	0.26	0.31	0.30	0.27	3.61
NahG-Désirée	MOCK	upper	1	0.00	0.07	0.00	0.03	0.01	1.10	1.08	3.29
NahG-Désirée	MOCK	upper	1	0.00	0.04	0.00	0.02	0.01	1.25	0.89	2.64
NahG-Désirée	MOCK	upper	1	0.00	0.04	0.00	0.03	0.01	1.33	1.28	4.51
NahG-Désirée	MOCK	upper	3	0.00	0.06	0.00	0.04	0.02	1.88	1.44	5.93
NahG-Désirée	MOCK	upper	3	0.00	0.08	0.01	0.07	0.05	1.19	0.98	3.97
NahG-Désirée	MOCK	upper	3	0.00	0.04	0.00	0.12	0.02	0.82	0.86	5.99
NahG-Désirée	MOCK	upper	4	0.00	0.04	0.00	0.01	0.01	1.29	1.22	4.84
NahG-Désirée	MOCK	upper	4	0.00	0.06	0.01	0.03	0.01	0.83	0.96	2.60
NahG-Désirée	MOCK	upper	4	0.00	0.04	0.01	0.08	0.02	0.67	0.83	3.51
NahG-Désirée	MOCK	upper	5	0.00	0.07	0.02	0.25	0.08	0.53	0.59	7.16
NahG-Désirée	MOCK	upper	5	0.00	0.03	0.02	0.04	0.02	0.92	1.23	3.13
NahG-Désirée	MOCK	upper	5	0.00	0.03	0.06	0.02	0.02	0.67	0.82	0.76
NahG-Désirée	MOCK	upper	7	0.00	0.06	0.06	0.34	0.12	0.31	0.53	4.81
NahG-Désirée	MOCK	upper	7	0.00	0.06	0.02	0.06	0.02	1.15	1.96	4.68
NahG-Désirée	MOCK	upper	7	0.00	0.07	0.02	0.24	0.11	0.58	0.83	6.63
NahG-Désirée	MOCK	upper	8	0.00	0.17	0.04	0.47	0.19	0.47	0.67	3.96
NahG-Désirée	MOCK	upper	8	0.00	0.02	0.01	0.07	0.03	0.64	0.53	1.95
NahG-Désirée	MOCK	upper	8	0.00	0.03	0.09	0.17	0.08	0.44	0.44	0.64
NahG-Désirée	MOCK	upper	9	0.00	0.10	0.02	0.59	0.12	0.44	0.61	8.67
NahG-Désirée	MOCK	upper	9	0.00	0.09	0.05	0.33	0.11	0.30	0.44	8.05
NahG-Désirée	MOCK	upper	9	0.00	0.07	0.04	0.30	0.08	0.35	0.42	3.04
NahG-Désirée	MOCK	upper	11	0.00	0.04	0.02	0.38	0.10	0.57	0.78	2.79
NahG-Désirée	MOCK	upper	11	0.00	0.04	0.01	0.22	0.08	0.30	0.44	1.22
NahG-Désirée	MOCK	upper	11	0.00	0.03	0.01	0.18	0.05	0.78	0.93	3.20
NahG-Désirée	PVY	inoculated	1	0.47	NA	4.57	0.03	0.04	0.31	0.40	0.16
NahG-Désirée	PVY	inoculated	1	1.02	0.05	1.35	0.10	0.07	0.25	0.40	0.57
NahG-Désirée	PVY	inoculated	1	0.26	0.03	0.55	0.09	0.05	0.71	1.01	1.02
NahG-Désirée	PVY	inoculated	3	0.54	0.29	0.41	0.97	0.19	0.44	0.22	0.84
NahG-Désirée	PVY	inoculated	3	0.67	0.09	0.13	0.55	0.07	0.37	0.33	1.15
NahG-Désirée	PVY	inoculated	3	0.40	0.09	0.11	0.36	0.13	0.58	0.51	2.48
NahG-Désirée	PVY	inoculated	4	NA	0.28	0.43	0.12	0.49	0.37	0.63	1.10
NahG-Désirée	PVY	inoculated	4	4.51	0.69	0.17	0.15	0.39	0.37	0.57	0.70
NahG-Désirée	PVY	inoculated	4	2.17	1.05	1.88	0.41	1.25	0.30	0.29	0.62
NahG-Désirée	PVY	inoculated	5	4.59	12.46	1.57	1.43	3.35	0.33	0.32	2.07
NahG-Désirée	PVY	inoculated	5	1.19	NA	0.52	0.36	0.93	0.46	0.58	1.04
NahG-Désirée	PVY	inoculated	5	3.88	5.02	0.52	0.79	2.28	0.65	0.76	1.82
NahG-Désirée	PVY	inoculated	7	8.93	13.14	4.62	2.16	5.38	0.74	0.53	1.54
NahG-Désirée	PVY	inoculated	7	15.49	4.99	2.74	2.77	1.37	0.04	0.03	0.10

Genotype	Inoculum	Leaf type	dpi	PVY	PR-1b	Glu-I	Glu-II	Glu-II	RA	CAB4	GBSSI
NahG-Désirée	PVY	inoculated	7	6.43	10.78	6.57	4.94	4.26	0.21	0.24	2.31
NahG-Désirée	PVY	upper	1	0.00	0.16	0.01	0.07	0.01	NA	2.02	1.83
NahG-Désirée	PVY	upper	1	0.00	0.04	0.03	0.09	0.02	0.83	1.23	2.39
NahG-Désirée	PVY	upper	1	0.00	0.02	0.04	0.01	0.01	0.90	1.44	1.06
NahG-Désirée	PVY	upper	3	0.00	0.03	0.01	0.03	0.02	0.64	1.49	1.27
NahG-Désirée	PVY	upper	3	0.00	0.03	0.04	0.06	0.02	1.01	1.79	2.34
NahG-Désirée	PVY	upper	3	0.00	0.18	0.04	0.27	0.33	0.81	1.79	2.51
NahG-Désirée	PVY	upper	4	0.00	0.03	0.01	0.01	0.01	0.61	1.41	2.75
NahG-Désirée	PVY	upper	4	0.00	0.10	0.03	0.02	0.02	0.92	1.54	1.68
NahG-Désirée	PVY	upper	4	0.00	0.04	0.03	0.03	0.03	0.67	1.20	1.90
NahG-Désirée	PVY	upper	5	0.00	0.06	0.02	0.10	0.04	0.80	NA	7.84
NahG-Désirée	PVY	upper	5	0.00	0.07	0.00	0.04	0.02	1.07	1.25	4.01
NahG-Désirée	PVY	upper	5	0.00	0.13	0.02	0.02	0.02	1.60	1.79	5.36
NahG-Désirée	PVY	upper	7	0.00	0.02	0.07	0.10	0.08	0.63	1.12	4.44
NahG-Désirée	PVY	upper	7	0.00	0.02	0.04	0.04	0.02	0.69	1.40	2.39
NahG-Désirée	PVY	upper	7	0.00	0.11	0.08	0.43	0.16	0.60	1.12	5.94
NahG-Désirée	PVY	upper	8	0.00	0.05	0.08	0.27	0.12	0.55	0.76	6.92
NahG-Désirée	PVY	upper	8	0.00	0.05	0.07	0.45	0.15	0.47	0.75	8.16
NahG-Désirée	PVY	upper	8	0.00	0.03	0.02	0.11	0.05	0.51	0.95	4.32
NahG-Désirée	PVY	upper	9	0.31	0.10	0.28	0.32	0.15	0.58	0.99	6.81
NahG-Désirée	PVY	upper	9	0.00	0.20	1.05	0.59	0.39	0.84	1.07	5.91
NahG-Désirée	PVY	upper	9	0.00	0.14	0.41	0.53	0.30	0.51	0.76	8.64
NahG-Désirée	PVY	upper	11	0.00	0.07	0.06	0.34	0.14	0.73	0.73	3.09
NahG-Désirée	PVY	upper	11	1.29	1.80	0.25	2.27	1.75	0.66	1.07	1.97
NahG-Désirée	PVY	upper	11	0.00	0.07	0.09	0.41	0.17	0.51	0.74	4.20
